# Use of outcomes to evaluate surveillance systems for bioterrorist attacks

**DOI:** 10.1186/1472-6947-10-25

**Published:** 2010-05-07

**Authors:** Kerry A McBrien, Ken P Kleinman, Allyson M Abrams, Lisa A Prosser

**Affiliations:** 1 Harvard School of Public Health, Boston, Massachusetts, USA; 2 Blue Cross Blue Shield of Massachusetts, Boston, USA; 3 Department of Population Medicine, Harvard Medical School and Harvard Pilgrim Health Care, Boston, USA; 4 University of Michigan Health System, Ann Arbor, Michigan, USA

## Abstract

**Background:**

Syndromic surveillance systems can potentially be used to detect a bioterrorist attack earlier than traditional surveillance, by virtue of their near real-time analysis of relevant data. Receiver operator characteristic (ROC) curve analysis using the area under the curve (AUC) as a comparison metric has been recommended as a practical evaluation tool for syndromic surveillance systems, yet traditional ROC curves do not account for timeliness of detection or subsequent time-dependent health outcomes.

**Methods:**

Using a decision-analytic approach, we predicted outcomes, measured in lives, quality adjusted life years (QALYs), and costs, for a series of simulated bioterrorist attacks. We then evaluated seven detection algorithms applied to syndromic surveillance data using outcomes-weighted ROC curves compared to simple ROC curves and timeliness-weighted ROC curves. We performed sensitivity analyses by varying the model inputs between best and worst case scenarios and by applying different methods of AUC calculation.

**Results:**

The decision analytic model results indicate that if a surveillance system was successful in detecting an attack, and measures were immediately taken to deliver treatment to the population, the lives, QALYs and dollars lost could be reduced considerably. The ROC curve analysis shows that the incorporation of outcomes into the evaluation metric has an important effect on the apparent performance of the surveillance systems. The relative order of performance is also heavily dependent on the choice of AUC calculation method.

**Conclusions:**

This study demonstrates the importance of accounting for mortality, morbidity and costs in the evaluation of syndromic surveillance systems. Incorporating these outcomes into the ROC curve analysis allows for more accurate identification of the optimal method for signaling a possible bioterrorist attack. In addition, the parameters used to construct an ROC curve should be given careful consideration.

## Background

Given the realistic possibility of bioterrorist attacks, a key public health challenge lies in identifying practical disease surveillance methods that will minimize associated casualties and costs by enabling a timely response. Illnesses caused by many bioterrorism agents, including anthrax, present with a prodrome indistinguishable from that of influenza or other common illnesses, making syndromic surveillance systems a useful option for the detection of bioterrorist attacks [1]. These systems differ from traditional public health surveillance methods, which rely upon reported disease-specific diagnoses and instead use statistical algorithms to detect aberrations in pre-diagnostic data. For example, cases of inhalational anthrax may manifest as an increase in the number of ICD-9 codes for bronchitis, cough, or pneumonia in an electronic medical record system [2].

Due to the paucity of authentic data on bioterrorist attacks, researchers have used simulated bioterrorist attacks to assess the performance of syndromic surveillance systems [3]. Previous studies have used the sensitivity, specificity, predictive values, and variations of receiver operating characteristic (ROC) curves to evaluate the performance of syndromic surveillance systems using simulated data [3]. The success of a surveillance system, however, will depend not only on whether the attack was detected, but also on the timeliness with which it was detected [4]. Kleinman et al simulated a set of hypothetical bioterrorist attacks with anthrax [2] and, using modified ROC curve analysis, evaluated seven detection algorithms by weighting the sensitivity measure by the time lag in detecting an attack. They found that both the absolute performance as well as the relative performance of the systems differed after timeliness was incorporated into the metric [4].

Although timeliness adds an important element to the sensitivity metric, it remains a proxy for key health and financial outcomes: deaths, illnesses, and costs. In a follow-up study, Kleinman et al weighted the sensitivity metric by the proportion of affected individuals and found again that the weighting changed the relative performance of the systems [5]. This study extends previous research by incorporating associated costs, lives lost and illness averted into the sensitivity metric of ROC curves. It accounts for the health and financial benefits of early detection, while also accounting for consequences of side effects of prophylaxis, adverse events from treatment, and the long-term sequelae of disease.

## Methods

We simulated a series of anthrax attacks and performed an evaluation of seven statistical detection algorithms applied to syndromic surveillance data. The evaluation employed weighted ROC curves that incorporate the following outcomes in order of increasing comprehensiveness: lives, quality-adjusted life years (QALYs), and costs. A decision-analytic approach was used to predict outcomes using data from the simulated attacks. Predicted outcomes were then used to construct outcomes-weighted ROC curves for each of the candidate metrics.

### Simulated Attack Data

We used two data sets: simulated attack data and observed surveillance system data. Full data on the simulations and observed data can be found in a previous publication; we offer a brief summary here [2]. The observed data are counts of respiratory complaints recorded in electronic medical records of the ambulatory encounters of approximately 250,000 patients in eastern Massachusetts. In the simulation data, an attack is assumed to be the result of the release of anthrax spores from a crop dusting airplane. The attack resulted in simulated cases, of which a proportion corresponding to the proportion of the total population included in the observed surveillance system were added to the surveillance system data, by zip code. We then assessed the evidence for the attack using various statistical algorithms described below. Since detection ability depends on the time of year and day of the week, we repeated this exercise with three separate simulated attacks on each day of 2003. We recorded the simulated number affected on each day. The total population in this region is approximately 2.5 million [2].

We recorded which of the simulated attacks were detected and on which day, by each of seven algorithms, using 11 different sensitivity thresholds. Three of the algorithms, Scan 1, Scan 3, Scan 7, used space-time scan statistic methods with maximum signal durations of one, three or seven days respectively [6]. Three others, GLMM 1, GLMM 3, GLMM 7 used a Poisson generalized linear mixed effects model with fixed one, three or seven day durations [7]. The last method used a time series approach [4]. The number of false positive signals that occurred over a one-year period for each of the seven methods and thresholds was calculated as the number of "detections" in the absence of simulated attacks.

### Decision analytic model

**Figure 1:**
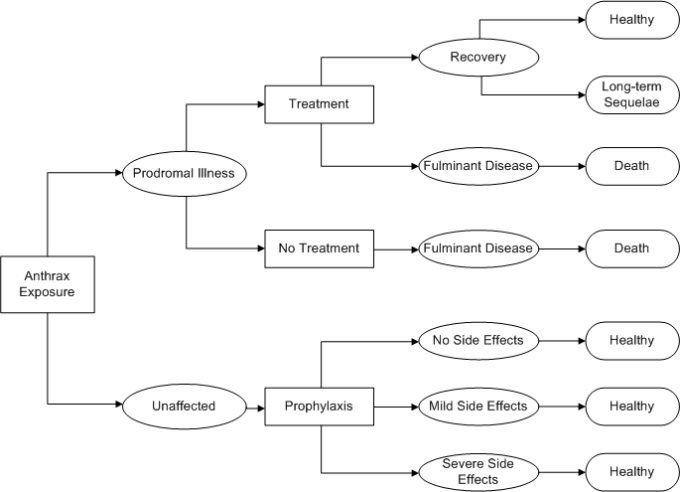
**Decision model**. Decision analytic model for a bioterrorist attack with *bacillus anthracis*.

Figure 1 depicts the states that an individual has the potential to progress through following a bioterrorist attack with anthrax. A healthy individual exposed to spores of bacillus anthracis may develop anthrax illness. The illness begins in the prodromal phase and, if not treated, will progress to the fulminant phase according to a time-dependent probability distribution [8]. Death was assumed to occur within 24 hrs of developing fulminant disease, regardless of whether medical treatment was provided [8]. Using the time-dependent probability of disease progression, we were able to estimate the number of individuals that would be expected to fall into each illness state (i.e., prodromal illness, fulminant disease, recovery with and without long-term sequelae, death) on each of days one through ten following a given attack.

If an attack was signaled by the detection system, it was assumed that all cases of anthrax would receive appropriate and timely medical care. Those with prodromal illness would be treated with multiple antibiotics, with regimens similar to those used for the anthrax attacks in the U.S. in 2001 [8]. If treated, an individual with prodromal illness has a chance of survival. Those who recover may experience sequelae of anthrax illness. Those with fulminant disease would be admitted to critical care units and treated, but were assumed to ultimately die from anthrax exposure.

The remainder of the population of Eastern Massachusetts would be considered at risk, and would be started on antibiotic prophylaxis with a 60-day course of oral ciprofloxacin. Prophylaxis was considered to be 100% effective in preventing the development of illness among those exposed to anthrax. A percentage of these individuals would be expected to experience mild or severe adverse effects from the medication. However, it was assumed that oral ciprofloxacin does not carry any risk of mortality.

### Decision Analysis Inputs

The model inputs were derived using a combination of literature review, empirical calculation, and expert interview. They are summarized in Table 1.

**Table 1:** Decision analytic model inputs

Variable	Value	Range for sensitivity analysis	Source
Health state transition probabilities			

Probability of recovery from fulminant anthrax	0	-	[8]
Probability of recovery from prodromal anthrax	0.857	0.66-0.9375	[8]
Probability of prophylactic antibiotic effectiveness	1.0	-	[9]
Probability of developing mild side effects from antibiotics	0.57	0.3-0.57	[10]
Probability of developing severe side effects from antibiotics	0.003	0-0.01	[10]
Probability of seeking medical attention for mild side effects	0.16	0-0.25	[10]
Probability of seeking medical attention for severe side effects	1.0	-	[10]

Health state utility values			

Death	0	-	
Recovery from anthrax	0.56*	0.4*-0.56^†^	[12,13,24],
Mild side effects from antibiotics	0.998	0.994-0.999	[10,14]
Severe side effects from antibiotics	0.992	0.980-0.997	[10]
No side effects from antibiotics - healthy	1.0	-	

Cost estimates (2006 USD)			

Cost of treatment of prodromal anthrax	9,223	9223-18446^‡^	[15-17],
Cost of a course of prophylactic antibiotics	638	-	[19,20]
Cost of office visit for mild side effects	30	-	[17]
Cost of treatment of severe side effects	189	-	[17,18]
Willingness to pay to avoid sequelae associated with recovery from anthrax	214,910^§ ^per year	Life*-5 yrs^†^	[12,22],
Value of a statistical life	7.3 m	5.51 m-13.23 m	[22]

*This health state utility was matched to the EQ-5D [24], and assumed to continue throughout the life span. An average age of 36.5 years was assumed according to the US Census Bureau and average remaining life span of 43 years was calculated using the 2002 US Life Tables [25,26].

^†^A five year duration for this health state utility was used as the upper bound.

^‡^The upper bound was estimated by doubling the cost for the base case.

^§^This estimate was adjusted to assume that one out of every six anthrax patients would be able to return to work.

### Analysis of Lives and Quality-Adjusted Life-Years (QALYs)

Three main endpoints were included in the analysis: lives, quality-adjusted life-years, and costs. The total number of deaths, given detection on a given day following an attack, was determined by summing the number of fulminant and dead cases and adding to this the number of prodromal cases expected to progress to the fulminant stage despite treatment. The probabilities of progressing through the states of anthrax illness were taken from a comprehensive review of anthrax illness [8]. The base case estimate is that associated with the 2001 US attacks, where six out of seven individuals with prodromal illness survived. The probabilities associated with prophylactic antibiotics were derived from CDC surveillance reports of the 2001 prophylaxis population [9,10]. Other probabilities were derived from the literature and expert opinion. (P. Brachman, pers. comm.)

To calculate the number of quality-adjusted life years gained through earlier detection, each final health state was assigned a utility value between zero and one, one being equivalent to perfect health and zero being equivalent to death. Utilities represent individuals' relative preference for a health state and are used to adjust for the lower quality of life associated with short- or long-term morbidity [11]. By adjusting years of life by their associated health state utilities, the number of quality adjusted life years (QALYs) can be calculated. One QALY can be roughly described as equivalent to one year in perfect health. This metric accounts for both life years gained due to averted deaths and morbidity averted due to earlier detection. The number of life years gained for an averted death was calculated using US life tables and an average population age of 36.5 years.

The long-term sequelae of anthrax illness include depression, anxiety and long-term cardiac and respiratory disability [12]. The base case estimate for the utility associated with this state assumes life-long sequelae [13]. The range for sensitivity analysis varies the duration of sequelae from 5 years to life. Side effects from antibiotics were assumed to last for three days in the base case and were varied from one to seven days in the sensitivity analysis. Health state utilities were derived from the literature [14]. An annual discount rate of 0.03 was applied to all future health states.

### Cost analysis

Defining outcomes in terms of costs allows for the most comprehensive adjustment as it takes into account morbidity and mortality, as well as cost of treatment. Costs of medical care were based on Medicare payment rates, wholesale prescription drug prices, and published estimates from health economic literature. The cost of treating prodromal anthrax included costs of hospitalization and infectious disease consultative services [15-17]. The cost associated with side effects is that of a brief office visit for those with mild side effects who seek treatment and is the cost of an emergency room visit for all those with severe side effects [17,18]. The cost of prophylactic antibiotics assumed a 60-day regimen of oral ciprofloxacin [19,20]. All costs were converted to 2006 US dollars [21].

In the cost analysis, health effects are converted to dollar values using published willingness-to-pay amounts and estimates for the value of a statistical life [12,22]. For example, the published value for willingness to pay to avoid permanent disability was $1,032 per day and this value would replace the health state utility value used in the QALY analysis [22]. Therefore, the cost analysis includes direct costs of medical care as well as morbidity and mortality effects converted into dollar values.

### Weighted ROC curves

We used weighted ROC curves to determine the relative performance of the detection algorithms. Traditional ROC curves are constructed by plotting the sensitivity versus 1-specificity for various decision thresholds of a test. A comparison of the area under the ROC curve (AUC) can be used to determine the relative performance of several different tests [4]. The weighted ROC curves used here replace the sensitivity of the test traditionally found on the y-axis of the ROC curve with a metric weighted by health outcomes.

In the case of disease surveillance, the benefit of detection depends on the number of adverse events averted, which in turn depends on the timeliness of detection. The surveillance system was considered to have failed if the attack was not detected before the tenth day, as it is generally accepted that an anthrax epidemic would be caught by traditional surveillance methods no later than the tenth day [4]. Using the expected outcomes on day nine as a baseline, we calculated the benefits of early detection by weighting each detected attack by the proportion of the outcome saved given the day of detection. The proportions of lives saved for each detected attack were then averaged together with undetected attacks, over all 1095 simulations, to determine a weighted sensitivity for each algorithm at each threshold. This same procedure was used to determine a weighted sensitivity for QALYs and costs saved.

The weighted ROC curves were constructed with the weighted sensitivity on the y-axis, and the false positive rate per day on the x-axis. The area under the weighted ROC curves (AUC) was calculated for each of the seven statistical algorithms across the three dimensions of lives, QALYs and costs. The relative performance of the seven statistical algorithms was assessed by comparing their respective AUCs. In the base case analysis, the AUC was calculated using a non-parametric trapezoidal method [23].

### Sensitivity Analysis

**Figure 2:**
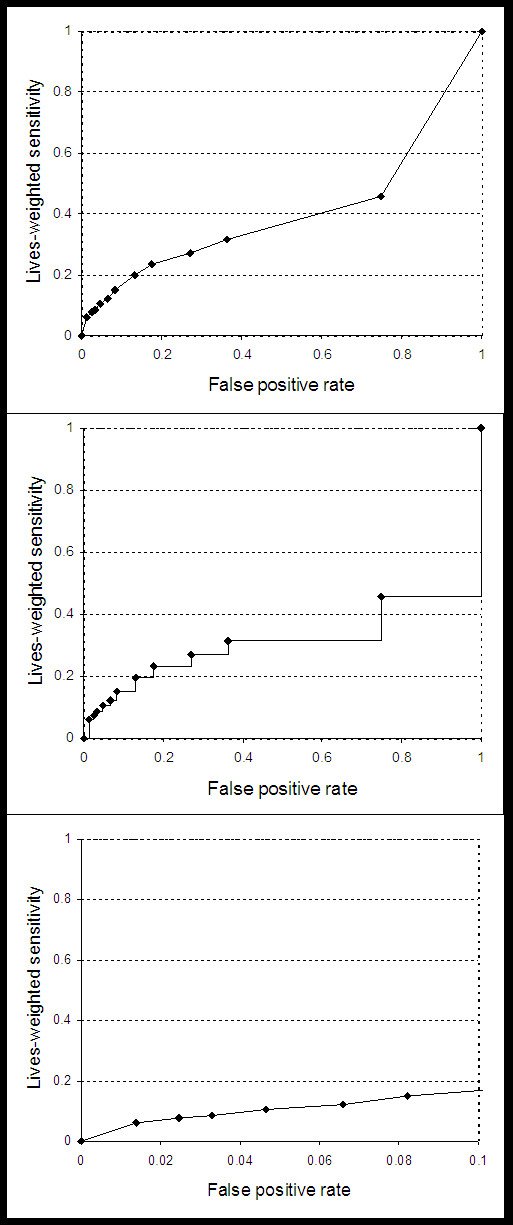
**AUC calculation methods**. Weighted receiver operator characteristic curve for the generalized linear mixed effects model (GLMM 1) using lives-weighted sensitivity, depicting three methods for determining the area under the curve (AUC). From top to bottom: trapezoidal, rectangular, and truncated.

Three types of sensitivity analyses were performed. First, input parameters were varied using the ranges identified in Table 1 to represent best and worst case scenarios. These scenarios reflect the parameter sets that bound the results for best and worst performance of the surveillance systems. Second, we reduced the proportion of the population required to receive prophylaxis, assuming it would be possible to accurately identify the location of the release, thus requiring prophylaxis for only 40% of the population. Third, we used alternate methods to calculate the AUC: a rectangular method and a truncated method. The rectangular method assumes that movement between test thresholds is discrete and that the sensitivity of the systems remains constant between false positive rates. The truncated method is used to reflect the fact that a high false positive rate would not be tolerated, and that only a portion of the curve is relevant. We used a false positive rate of 0.1 alarms per day as a cut-off point. Figure 2 shows a graphical depiction of the three AUC methods.

## Results

### Lives, QALYs, and Costs by Day of Detection

**Figure 3:**
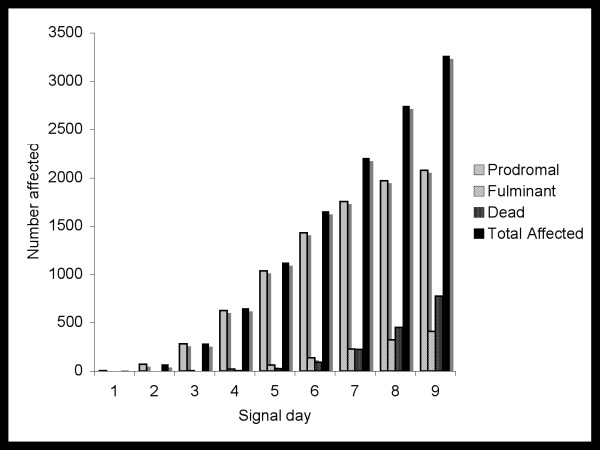
**Time dependent illness counts**. Number predicted to have Anthrax disease by phase of illness and day of detection following a bioterrorist attack with *bacillus anthracis*.

**Figure 4:**
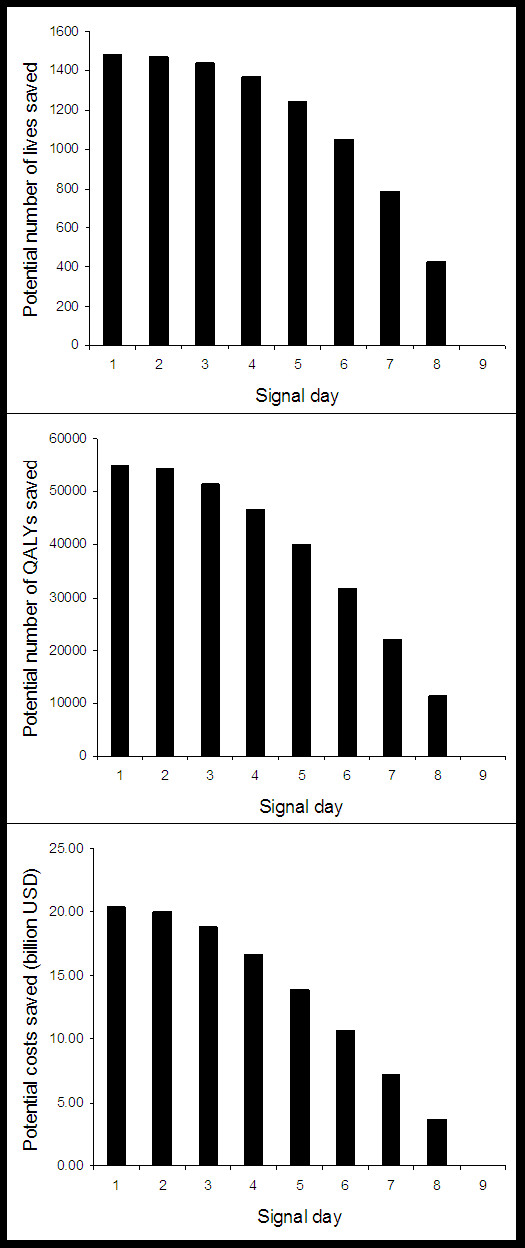
**Time-dependent attack outcomes**. Potential lives, quality adjusted life-years (QALYs), and costs saved by day of detection following a bioterrorist attack with *bacillus anthracis*.

Figure 3 shows the number of people predicted to have each of the three phases of Anthrax illness on Days 1 through 9 following an attack. The remainder of the population at risk would be eligible for prophylaxis. Of note, the increase in the total number of people affected each day follows a non-linear pattern. Figure 4 depicts the number of lives, QALYs and costs that we predict could be saved by day of detection. Again, the change by day is non-linear, indicating that a one-day delay in detection has a differential impact depending on the number of days that have elapsed since the attack. For instance, a delay from day 4 to day 5 would result in a larger loss than a delay from day 1 to day 2. Detection in the first three days has a similar effect; in this case our model estimates that approximately 1400 lives, 50,000 QALYs, and $18 billion USD could potentially be saved (Figure 3).

### Weighted ROC Curve Analysis - Base case

Using weighted ROC curves that incorporate lives saved, QALYs gained, or costs, the Time-series system is consistently the best-performing method. GLMM7 consistently performed worst and GLMM1 is second best. The relative performance of other tests varies by the measure used. (Table 2) These areas were calculated using the trapezoidal method, which assumes that the sensitivity of the detection algorithms is continuously modifiable. In some cases the curves required extrapolation to a false positive rate of 1 alarm per day- in these cases the extrapolation point chosen was (1,1).

**Table 2:** AUC of weighted ROC curves - base case

Lives - weighted		QALYs - weighted		Costs - weighted		**Timeliness - weighted **[4]	
**System**	**AUC**	**System**	**AUC**	**System**	**AUC**	**System**	**AUC**

TS	0.514	TS	0.448	TS	0.424	TS	0.65
GLMM 1	0.447	GLMM 1	0.411	GLMM 1	0.397	Scan 1	0.41
Scan 1	0.431	Scan 1	0.36	GLMM 3	0.336	Scan 3	0.378
Scan 3	0.413	GLMM 3	0.356	Scan 1	0.333	Scan 7	0.349
GLMM 3	0.409	Scan 3	0.34	Scan 3	0.313	GLMM 3	0.276
Scan 7	0.377	Scan 7	0.309	Scan 7	0.283	GLMM 7	0.271
GLMM 7	0.316	GLMM 7	0.269	GLMM 7	0.251	GLMM 1	0.235

### Weighted ROC Curve Analysis - Sensitivity analysis

In the sensitivity analysis that assumes the most optimistic scenario, there is little change in the relative performance of the candidate methods. Time-series remains the best-performing and GLMM7 is consistently the worst. (Table 3) The relative order of performance does change for the worst case scenario and Time-series is no longer consistently the best-performing method. (Table 4)

**Table 3:** Sensitivity analysis of AUC of weighted ROC curves - best case scenario

Lives - weighted		QALYs - weighted		Costs - weighted	
**System**	**AUC**	**System**	**AUC**	**System**	**AUC**

TS	0.540	TS	0.499	TS	0.533
Scan 1	0.470	GLMM 1	0.432	GLMM 1	0.469
GLMM 1	0.468	Scan 1	0.406	Scan 1	0.469
Scan 3	0.454	GLMM 3	0.385	Scan 3	0.455
GLMM 3	0.438	Scan 3	0.384	GLMM 3	0.439
Scan 7	0.413	Scan 7	0.348	Scan 7	0.415
GLMM 7	0.339	GLMM 7	0.293	GLMM 7	0.340

**Table 4:** Sensitivity analysis of AUC of weighted ROC curves - worst case scenario

Lives - weighted		QALYs - weighted		Costs - weighted	
**System**	**AUC**	**System**	**AUC**	**System**	**AUC**

TS	0.467	GLMM 1	0.375	GLMM 1	0.329
GLMM 1	0.434	TS	0.321	GLMM 3	0.242
Scan 1	0.400	GLMM 3	0.306	TS	0.212
Scan 3	0.391	Scan 1	0.273	Scan 1	0.178
GLMM 3	0.385	Scan 3	0.263	Scan 3	0.170
Scan 7	0.352	Scan 7	0.240	GLMM 7	0.168
GLMM 7	0.300	GLMM 7	0.225	Scan 7	0.155

When the population targeted for prophylaxis is reduced to 40% of the population in the base case, the relative ordering of performance remains consistent with that of the base case. (Table 5)

**Table 5:** Sensitivity analysis of AUC of weighted ROC curves - 40% prophylaxis

Lives - weighted		QALYs - weighted		Costs - weighted	
**System**	**AUC**	**System**	**AUC**	**System**	**AUC**

TS	0.514	TS	0.467	TS	0.449
GLMM 1	0.447	GLMM 1	0.415	GLMM 1	0.403
Scan 1	0.431	Scan 1	0.371	Scan 1	0.348
Scan 3	0.413	GLMM 3	0.363	GLMM 3	0.345
GLMM 3	0.409	Scan 3	0.351	Scan 3	0.327
Scan 7	0.377	Scan 7	0.318	Scan 7	0.296
GLMM 7	0.316	GLMM 7	0.275	GLMM 7	0.259

When the AUC calculation method is changed from trapezoidal to either rectangular or truncated, there is marked difference in the relative performance of the surveillance systems, as is demonstrated in Tables 6 and 7. While GLMM 7 remains the worst performer, the two systems that consistently had the top performances with the trapezoidal calculation, Time-series and GLMM 1, have relatively poor performance using either the rectangular or the truncated method. The Scan systems appear to have the best performance using these methods, with Scan 3 being the best performer across all analyses. It is also notable that the ordering of performance is somewhat more consistent across the three outcome weightings using either the rectangular or truncated method, as compared to the trapezoidal method.

**Table 6:** Sensitivity analysis of AUC of weighted ROC curves - rectangular calculation method

Lives - weighted		QALYs - weighted		Costs - weighted	
**System**	**AUC**	**System**	**AUC**	**System**	**AUC**

Scan 3	0.364	Scan 3	0.297	Scan 3	0.272
Scan 7	0.347	Scan 7	0.282	Scan 7	0.258
Scan 1	0.325	Scan 1	0.267	Scan 1	0.245
GLMM 3	0.306	TS	0.254	TS	0.235
TS	0.303	GLMM 3	0.248	GLMM 3	0.227
GLMM 1	0.260	GLMM 1	0.213	GLMM 1	0.195
GLMM 7	0.246	GLMM 7	0.199	GLMM 7	0.182

**Table 7:** Sensitivity analysis of AUC of weighted ROC curves - truncated method

Lives - weighted		QALYs - weighted		Costs - weighted	
**System**	**AUC**	**System**	**AUC**	**System**	**AUC**

Scan 3	0.205	Scan 3	0.163	Scan 3	0.148
Scan 1	0.201	Scan 1	0.162	Scan 1	0.148
Scan 7	0.171	Scan 7	0.133	Scan 7	0.119
GLMM 3	0.104	GLMM 3	0.082	TS	0.075
TS	0.097	TS	0.081	GLMM 3	0.074
GLMM 1	0.065	GLMM 1	0.052	GLMM 1	0.047
GLMM 7	0.041	GLMM 7	0.032	GLMM 7	0.029

## Discussion

Through the use of decision analytic modeling, we were able to translate the number of people affected by a hypothetical bioterrorist attack to relevant outcomes and incorporate these outcomes into the evaluation metric. The outcomes were lives lost, QALYs lost, and costs incurred, costs being the most comprehensive of the three. In the base case, using the trapezoidal method of AUC calculation, the relative order of performance remained fairly consistent across the three outcome weights. However, we found the relative order of performance was sensitive to the model inputs as well as the method of AUC calculation.

Our model predicts that, if undetected until the ninth day, a bioterrorist attack with *bacillus anthracis *would have a significant detrimental effect on the health of the population. If a surveillance system was successful in detecting the attack before the ninth day, and measures were immediately taken to deliver treatment to the population, the lives, QALYs and dollars that would be lost could be reduced considerably. Earlier detection results in better outcomes: our model estimates an absolute cost savings of several billion dollars for a detection on the eighth day rather than the ninth. Conversely, a false positive alarm has negative consequences associated with the unnecessary use of prophylactic antibiotics, namely the cost incurred and the adverse effects of the medication. Consequently, a high-performing surveillance system should not only be capable of detecting an attack before the ninth day, but should also detect the attack in as timely a manner as possible and with a low rate of false positives.

Public health authorities must consider both the positive and negative aspects of the programs they choose to implement. In the case of surveillance for bioterrorism attacks, the benefits of early detection must be balanced by the adverse effects of false positive alarms, an aspect of surveillance systems that supports the use of weighted ROC curve analysis in their evaluation. Results comparing alternative surveillance algorithms could be used to select an optimal algorithm depending on the outcome public decision makers choose to optimize. This kind of information, and the cost information assembled in Table 1, can help inform discussions about the value and appropriate role for syndromic surveillance.

When timeliness was incorporated into the evaluation metric by Kleinman et al [4], the Time-series method was the best performer, consistent with our results. The order of relative performance of the other systems however was different in the present analysis. Using a different weighting scheme, Kleinman et al also performed an evaluation of the systems that incorporated the number of people affected by the attacks [5], and found an order of relative performance that differs from both their previous analysis as well the present analysis. As noted in the results, the impact of a delay in detection varies with day of detection. We have shown that this variation also affects the apparent performance of the surveillance systems, and thus the incorporation of outcomes into the evaluation metric has an important effect on their ranking.

It is reasonable to conclude that the shape of the outbreak also plays a part in the relative order of performance. If the increase in the number affected is greater during the first days following the attack, it follows that a detection system that performs best in those first days would result in fewer losses. However, if the increase in the number of affected people is highest several days after the attack, a detection system with a higher cumulative sensitivity during the preceding days would have the best performance, even if detection is delayed by several days. Regardless of the shape of the outbreak, in all but a linear relationship between number affected and time, the incorporation of outcomes has a significant impact on relative performance. The modified ROC curves described in this paper allow for several dependent variables to be taken into account in one evaluation metric.

The results remained fairly constant in the 'best' and 'worst' case scenario analyses, indicating that our model is robust to variation in model inputs. However, the relative order of performance is heavily dependent on the choice of AUC calculation method. The rectangular and truncated methods produced results quite different from the classic trapezoidal method. The trapezoidal method assumes that the surveillance system threshold can be adjusted in a continuous manner, such that the false positive rate can be set anywhere between zero and one. This may not in fact be a reasonable assumption given the complexity underlying the statistical algorithms used by the surveillance systems. Moreover, due to the need for extrapolation and the resulting shape of the curves (Figure 2), this method gives more weight to the latter portion of the curve where there are fewer data points. The rectangular method assumes that the thresholds are discrete and that the sensitivity of each detection algorithm has a preset maximum. The area is therefore limited by the preset maximum and the relative performance as measured by the AUC is thus affected. The truncated method goes a step further and assumes that there is a false positive rate beyond which the negative consequences are too great to consider using the system, allowing the remainder of the graph to be disregarded. In this case, we arbitrarily chose 0.1 false positives per day as the cutoff. As is demonstrated in the results, the relative ordering changed significantly from that determined in the base case.

Further research into the differences between the methods of area calculation is needed. If these evaluation methods were adopted and used by public health authorities, consideration should be given to the assumptions underlying the method of ROC curve construction, including the shape of the outbreak, the flexibility of the detection algorithms and the threshold for an acceptable false positive rate. For example, if an acceptable false positive rate were defined, this would restrict the portion of the curve to be studied and potentially minimize the variation when alternative calculation methods are used. The relative performances of the surveillance systems within these bounds would be more accurately applicable to a real-world setting.

Furthermore, the interpretation of these weighted ROC curves is limited due to the nature of the data used to construct the axes, a constraint shared by earlier analyses of this type [4]. The false-positive rate on the x-axis is based on only one year of historical data while the sensitivity is calculated from a simulated data set with multiple events that occur an arbitrary number of times. Rather than using the analysis to draw conclusions about the absolute performance of each system, the intention is to compare the area under the weighted ROC curves from the seven statistical algorithms in order to assess their performance relative to each other.

Although the probability and utility estimates were the best estimates available from the literature, we had limited data on some model inputs due to the limited number of anthrax cases. For example, the probability of recovery from anthrax disease was based on the only data available, the reported case series of seven individuals treated for prodromal anthrax in the 2001 outbreak [8]. Furthermore, the analysis assumed that all individuals with symptomatic anthrax illness would be treated on the day of detection and that antibiotic prophylaxis would be provided within one day to all persons at risk, an idealized scenario that may not be met in practice.

## Conclusion

This study demonstrates the importance of accounting for mortality, morbidity, and costs. Incorporating these outcomes into the ROC analysis allows for more accurate identification of the optimal method for signaling a possible bioterrorist attack. Future research should consider the capabilities of current surveillance systems and determine acceptable false positive rates, in order to appropriately calibrate available surveillance systems.

## Competing interests

The authors declare that they have no competing interests.

## Authors' contributions

KM participated in the design of the study, carried out the decision model and ROC curve analysis, and drafted the manuscript. KK designed the simulations and initial ROC curve analytic studies and helped conceive of the present study. AA carried out the simulations and the initial ROC curve analysis. LP conceived of the study, and participated in its design and coordination and helped to draft the manuscript. All authors read and approved the final manuscript.

## Pre-publication history

The pre-publication history for this paper can be accessed here:

http://www.biomedcentral.com/1472-6947/10/25/prepub
